# Monitoring the decomposition of wealth-related inequality in the use of regular antenatal care in Egypt (1995–2014)

**DOI:** 10.1186/s12889-020-09412-y

**Published:** 2020-08-27

**Authors:** Zeinab Khadr

**Affiliations:** 1 Department of Statistics, Faculty of Economics and Political Science, Cairo University, Giza, Egypt; 2 The Social Research Center, The American University in Cairo, Cairo, Egypt

**Keywords:** Egypt, Antenatal care, Concentration index, Decomposition, Wealth inequality, Blinder-Oaxaca decomposition

## Abstract

**Background:**

Between 1995 and 2014 Egypt successfully increased the use of regular antenatal care (URAC) among women from 30.4 to 82.9%. The same period saw a decrease in the wealth-based inequality in URAC. This paper investigates the changes in the main determinants contributing to the wealth-based inequality in URAC for the 2 years of 1995 and 2014, and the determinants that underlined the declines in this inequality.

**Methods:**

The secondary analysis was based on data from the 1995 and 2014 rounds of the Egypt Demographic and Health Survey. Logistic regression was implemented to model URAC for the 2 years and inequality was measured using the concentration index. Decomposition of the concentration index and Blinder -Oaxaca decomposition were implemented to assess the contribution of the URAC determinants to its inequality and the changes between 1995 and 2014.

**Results:**

Decomposition of inequalities in URAC in 1995 and 2014 showed that social determinants were the main contributors to these inequalities. More than 90% of the inequalities were explained by the living in rural Upper Egypt, women and their husbands secondary and higher education, the household standard of living, and birth order. These same determinants were responsible for more than 76% of the decline in the inequality in URAC between 1995 and 2014. Wide spread of poverty in rural Upper Egypt was found to contribute significantly to the inequality in URAC. Women and their husbands who have secondary or higher education maintained their high odds of URAC.

**Conclusion:**

Since poverty in rural Upper Egypt, and inequality in education and parity are crucial social determinants of URAC inequality and its change overtime, new policies and interventions need to focus not only on the health system but on social initiatives with an equity lens to tackle the structural causes underlying these factors and their inequalities.

## Background

Over a period of 25 years, Egypt succeeded in decreasing its maternal mortality ratio by more than 68.9%, from 106 deaths per 100,000 live births in 1990 to 33 deaths per 100,000 live births in 2015 [1, 2]. Campbell and colleagues attributed this success to the adoption of safe motherhood initiatives in Egypt [3]. These initiatives were based on the results of the 1992 and 2000 national maternal mortality surveys, which revealed that one fifth of maternal deaths were attributed to avoidable risk factors [3]. A lack of antenatal care, or the poor quality of this care where available, were responsible for almost one third of these deaths [3, 4]. Consequently, all safe motherhood initiatives in Egypt incorporated activities to promote the use of antenatal care [3]. These initiatives paid back in an increased use of antenatal care services by pregnant women. Data from the Egypt Demographic and Health Survey showed that, in 1995, only 39.1% of women with births in the 5 years preceding the survey had at least one antenatal care visit, and only 28.3% had regular antenatal care (four visits or more [5]) [6]. By 2014, women who had ever used antenatal care and those who had regularly used antenatal care had increased to 90.3 and 82.8%, respectively [7]. This relative improvement in the use of antenatal care services was also associated with a relatively moderate decline in wealth-based inequality. While the absolute difference between the richest and the poorest wealth quintile was almost 61 percentage points in 1995 for both ever receiving antenatal care and for regular antenatal care, it decreased to 12.3 percentage points for ever receiving antenatal care and 21.1% for receiving regular antenatal care in 2014 [6, 7].

The improved use of antenatal care and the reduction in wealth-based inequality in relation to it are associated with significant changes on the social and health fronts in Egypt in this period. On the social front, despite a 60% increase in population size between 1996 and 2016, Egypt managed to increase its GDP per capita by a factor of 3.5, from $944.2 in 1995 to $3477.9 in 2016. In addition, illiteracy eradication efforts succeeded in decreasing illiteracy rates by ten percentage points in 20 years to reach 29% in 2017 [8]. This decline in illiteracy was greater among women, among whom the illiteracy rate decreased by 13 percentage points to 37% in 2017. This improvement was also reflected in declines in those with low educational attainment and increases in higher educational attainment between 1995 and 2014. According to the data, 35% of the female population had no education in 1995, while in 2014, 16.5% of the same population had completed secondary or higher education [6]. In 2014, about 24.7% of the female population had no education and the percentage of those with completed secondary or higher education had almost doubled to reach 33.9% [7].

On the reproductive health front, in its implementation of the 1994 International Conference on Population and Development (ICPD) Programme of Action, Egypt focused on improving both accessibility and use of reproductive health services in Egypt. Access to reproductive health services was expanded to underserved women, in particular those residing in remote areas and in rural Upper Egypt [9]. This was achieved through the renovation of almost all of the Ministry of Health health units and clinics and by adding more than 300 mobile clinics to provide reproductive health to remote areas in Egypt. In addition, the ministry’s efforts were supported by many nongovernmental organizations, who ran their own family planning and reproductive health clinics under the ministry’s technical supervision and with its logistic support [10]. During the same period, reproductive health promotion campaigns played a significant role in promoting many messages regarding the importance of antenatal care, safe delivery, breastfeeding, immunization and premarital medical examinations. These campaigns were supported by the provision of training courses to media professionals on population and reproductive health issues [11].

In conclusion, there is no doubt that Egypt has achieved many successes on the social and reproductive health fronts. The two main questions posed by the current paper are: to what extent have these achievements contributed to tackling wealth-related inequalities in the use of regular antenatal care and its determinants, and how have the changes in the inequalities in these determinants contributed to the changes in the inequalities in the use of antenatal care?

### Determinants and inequality of antenatal care in Egypt

Analyses of the main determinants of maternal health services in Egypt, including the use of antenatal care, have received significant attention from researchers. Some research efforts have focused on understanding the main determinants of any use or of regular use of antenatal care. As in the literature on determinants of antenatal care use in developing countries [12–33], research in Egypt has confirmed the importance of both social and health needs as determinants of antenatal care [14, 18, 19, 25, 27]. On the social side, women’s age or age at birth, their education, their husbands’ education level, the women’s work status, the household standard of living/wealth, and place of residence, assessed in terms of regional level or by rural/urban differentiation, were identified as significant determinants of antenatal care [12–29, 31–34]. On the health dimension, most research highlighted the significant contribution of parity/birth order, previous birth experience, attributes of current pregnancy (in particular preceding birth interval), the survival status of the previous birth, and the experience of a terminated pregnancy [12–29].

Other research efforts focused on monitoring changes in the use of antenatal care over time [27, 29]. Modeling any use of antenatal care separately for the years 2000 and 2014, Zaky and colleagues showed that wealth, higher education attainment of both women and their husbands, and residence in frontier governorates were significantly related to the use of any antenatal care in 2000 [29]. However, most of these determinants had lost their significance, in particular education and regional differences, by 2014.

Recently, since the launch of the Commission on Social Determinants of Health in 2005 [34], many research efforts have focused on assessing inequality in maternal health services in developing countries. These efforts used different approaches in measuring inequality, including modeling the different factors that affect the use of maternal health services for the different social groups [30] and identifying the main determinants for each social category. Other research efforts have implemented a summary measure of inequality for different maternal health measures, in particular the concentration index and its decomposition to the contribution of their determinants including use of antenatal care [18, 31–33].

Inequalities in antenatal care use have received significant attention from researchers in Egypt. Some research has been limited to assessing inequality using the concentration index across wealth quintiles [32, 33] and educational attainment categories [32]. Other efforts have assessed inequality through marginal probabilities of use of antenatal care across two latent variables of socio-cultural resourcefulness and economic resourcefulness [18]. Findings from this research showed both socio-cultural resourcefulness and economic resourcefulness were positively related to use of antenatal care services in Egypt. The probability of seeking any antenatal care increases from 55.2 to 62.0% as women move from the lowest to the highest level of socio-cultural resourcefulness. Furthermore, the probability of regular antenatal care also increases from 63.1 to 70.3%, with similar movement among antenatal care users. Similarly, the probability of any antenatal care increases from 52.1 to 64.0% as women move from the lowest category to the highest category of economic resourcefulness.

This paper aims to investigate the changes in the main determinants contributing to the wealth-based inequality in URAC for the 2 years of 1995 and 2014, and the determinants that underlined the declines in this inequality. It also expands the analysis by decomposing the wealth-based inequality in the use of antenatal care and assessing the contribution to that inequality of the different determinants. It further decomposes the changes in wealth-based inequality to assess the contribution of the changes in its determinants inequalities using Blinder-Oaxaca decomposition. This analysis provides an insight into the contribution of the changes in the two groups of determinants (social and health needs) to the decline of wealth-based inequality in the use of antenatal care. The results of this analysis can support the ongoing efforts to tackle inequality in maternal health care through the identification of the policies needed to achieve the largest decline in these inequalities, as well as the appropriate points of intervention.

## Methods

### Data sources and study variables

The current study uses data from 2 years of the Egyptian Demographic and Health Surveys (1995 and 2014) to monitor the changes in antenatal care use and its determinants over this period of time. The Egyptian Demographic and Health Surveys are comprehensive reproductive health surveys that aim to provide detailed information on maternal and child health. Eight rounds have been conducted in Egypt over the last 25 years (in 1989, 1992, 1995, 2000, 2003, 2005, 2008, and 2014). The periodicity of these surveys establishes them as a significant and rich data repository for monitoring changes in levels of inequality in various dimensions of women’s reproductive health. The current research focuses on two rounds, namely the 1995 and 2014 rounds. This period is a significant one; during this time, Egypt adopted the International Conference on Population and Development’s (ICPD) Programme of Action and committed to and aimed to fulfill the Millennium Development Goals.

The study sample in this research was limited to currently married women who had had at least one live birth in the 5 years preceding the survey. The final analytical samples were 7532 women in 1995 and 10,864 women in 2014. These women account for 50.1 and 43.6% of the total sample of ever-married women in the surveys from 1995 and 2014, respectively.

Use of antenatal care in the current study is assessed in terms of use of regular antenatal care (URAC). URAC was assessed in terms of a dichotomous variable defined as: (1) having had at least four antenatal care visits during pregnancy; and (0) otherwise. The equity stratifier used in the current study was the wealth index. The wealth index is a commonly used measure of the economic status of the households in which women live. It is based on the first principle component produced in the factor analysis in a set of indicators regarding the physical characteristics and possession of consumer durable goods by the respondent’s household [35]. Of the two rounds used in the current study the wealth index was only available in 2014. As a result, the wealth index for the data from the EDHS 1995 was calculated using the Principal component analysis on the same list of physical characteristics and consumer durable goods reported by Macro International for the production of the wealth index [30, 31].

Determinants of antenatal care were classified into two main categories of variables: social determinants and health needs determinants. The social determinants included in the analysis are: 1) the respondent’s age at the birth of the child, measured in terms of five-year age groups (15–19, 20–24, 25–29, 30–34, 35–39, and 40+); 2) the respondent’s education, measured in four categories (no education, primary, secondary or higher education); 2) the husband’s education, measured in four categories (no education, primary, secondary or higher education); 3) the household standard of living, assessed in terms of having modern toilet facility (modern toilet with a flush), with lack of modern toilet facility used as a proxy for being poor; and 4) region (urban governorates, urban Lower Egypt, rural Lower Egypt, urban Upper Egypt and rural Upper Egypt).

Health needs are measured in terms of two groups of variables. The first relates to the current pregnancy, namely the length of the preceding birth interval (preceding birth intervals > 24 months = 1, otherwise = 0), having multiple births (single birth = 1, otherwise = 0) and birth order. The second group of variables relates to previous birth experience, namely delivery of previous birth in a medical care unit (yes = 1, no = 0), pervious birth by cesarean section (yes = 1, no = 0), previous birth was alive (yes = 1, no = 0). In addition, an experience of a terminated pregnancy (yes = 1, no = 0).

Weights provided by the EDHS datasets were used in the analysis to account for the under- or over-sampling of some geographical regions relative to others in the two datasets used in the analysis.

### Statistical analysis

The current research aims to monitor changes in wealth inequality in the use of antenatal care and its determinants in the period from 1995 to 2014. The antenatal care inequality in the current study was measured using the concentration index.

#### The concentration index

The concentration index is a fairly standard measurement tool for assessing inequality in health and health care [36–39]. It allows the measurement of health inequality while taking into consideration the distribution of the health variables across all categories of the equity stratifier.

For micro-level data, a convenient formula for the concentration index is defined as follows:

$$ \mathrm{CI}=\frac{2}{\upmu}\operatorname{cov}\ \left(\mathrm{h},\mathrm{r}\right), $$

where “cov” is the covariance between the health variable (h) and the fractional rank of the stratifying variable distribution. The concentration index varies between − 1 and 1 with zero indicating equality and negative values indicating disproportionate concentration of the health issue among the lowest social stratum compared to the other social strata of the population. In other words, a negative value of the concentration index for “bad” health (ill health) indicates that lower social groupings are more afflicted by this “bad” health.

Since the URAC variable in the current analysis is a binary variable, the current analysis adopts the Erreygers’ normalized concentration index which can be obtained as [40].

$$ {E}_i=\frac{4\ {\mu}_h CI}{\left(b-a\right)} $$

Where *μ*_*h*_ is the weighted average of the URAC and b and a indicate the upper and lower bounds of the URAC, respectively.

#### Decomposing the concentration index

Decomposition of the health concentration index into the contributions of its main determinants requires the specification of a linear additive regression model of the health measure (hi) on its determinants such as:

$$ {\mathrm{h}}_{\mathrm{i}}=\upalpha +\sum \limits_{\mathrm{k}.\mathrm{i}}{\upbeta}_{\mathrm{k}}{\mathrm{x}}_{\mathrm{k}\mathrm{i}}+{\upvarepsilon}_{\mathrm{i}}, $$

where the “xk” variables are health determinants and ε is a disturbance term. Given the linear regression model, the concentration index for h, CI, can be written as:

$$ \mathrm{CI}=\sum \limits_{\mathrm{k}}\frac{\left({\upbeta}_{\mathrm{k}}{\overline{\mathrm{x}}}_k\right){\mathrm{C}}_{\mathrm{k}}}{\upmu}+\frac{\mathrm{G}{\mathrm{C}}_{\mathrm{e}}}{\upmu}, $$

where μ is the mean of h_i_, ${\overline{\mathrm{x}}}_k$ is the mean of *x*_*k*_, *C*_*k*_ is the concentration index for *x*_*k*_ and GCe is the generalized concentration index for ei. In other words, the concentration index of a health measure can be divided into two main components, namely the estimated health inequalities due to inequalities in the investigated determinants, and a residual component, which captures the inequality in health that is not explained by the inequalities in these determinants.

The predicted health inequality can be simply defined as the weighted sum of the inequality in each of its determinants:

$$ \hat{\mathrm{C}\mathrm{I}}=\sum \limits_{\mathrm{k}}{\hat{\upeta}}_k{\mathrm{C}}_{\mathrm{k}} $$

The weights in this case are defined as the elasticity of the health determinant (x_k_):

$$ {\hat{\upeta}}_k=\frac{\left({\hat{\beta}}_k{\overline{\mathrm{x}}}_k\right)}{\upmu}, $$

Accordingly, for each determinant, its contribution to inequality is conceptually divided into two components: 1) the impact of the health determinant on the health measures assessed by the health elasticity (η_k_), in particular the value of the ${\hat{\beta}}_k$; and 2) the health determinant’s unequal distribution across the socioeconomic stratifier, assessed in terms of its concentration index (C_k_).

To identify the contribution of each health determinant (in percentage points) to the predicted health issue’s inequality, the partial contribution of each health determinant is divided by the $\hat{\mathrm{CI}}$ and multiplied by 100:

$$ \% contribution\ of\ determinant\ (K)=\frac{{\hat{\upeta}}_k{\mathrm{C}}_{\mathrm{k}}}{\hat{\mathrm{C}\mathrm{I}}}X100=\frac{{\hat{\upeta}}_k{\mathrm{C}}_{\mathrm{k}}}{\sum \limits_{\mathrm{k}}{\hat{\upeta}}_k{\mathrm{C}}_{\mathrm{k}}\ }X\ 100 $$

This % contribution can be interpreted as the proportion of decline (if k% is positive) or increase (if k% is negative) in the inequality of the health variable, if the K determinant would become equally distributed among the socioeconomic groups or the regression coefficient of the the determinant K would become zero.

It is important to point out that the decomposition of CI relies on the linearity of the relationship between the health measure and its determinants. However, since URAC is measured as dichotomous measures, which ideally implies the use of nonlinear regression models, this requires a decision as to whether to approximate the model by a linear regression using a linear probability model (LPM) or to approximate the decomposition of the CI [39]. Approximating the decomposition of CI can be done through the implementation of the marginal effects of the nonlinear models [36]. The marginal effect of the logit models (dh/dx) provides the change in the predicted probability, with a change of one unit in the explanatory variable controlling for the effects of other explanatory variables [39]. In another words, marginal effects provide an approximate assimilation of the linear regression for the nonlinear models, which allows the decomposition of the concentration index for dichotomous variables. The marginal effect of an explanatory variable has the same sign as its coefficient in the logit regression. Also, it has the same interpretation as the coefficient in the linear regression, where a positive sign indicates an increase in the probability of the health measure, a negative sign indicates a decrease in the probability and a large value indicates a major effect on the probability.

#### Decomposing the changes in inequality between 1995 and 2014

In the process of monitoring health inequality, more insights can be gained by identifying the contribution of each health determinant to the change in the concentration index over time. This contribution can be further divided into contribution due to: a) the change in the association between the determinant and the health measure over time; and b) the change in the inequality of this determinant over the same period of time. The Blinder-Oaxaca method is one of the most commonly used approaches in defining these two types of contributions [41–43]. The Blinder-Oaxaca method has been used extensively in the economic literature on wage discrimination in the labor market. The World Bank also implemented Blinder-Oaxaca in analyzing health inequalities between two social groups [43]. The current paper uses Blinder-Oaxaca in decomposing the changes in the concentration index between 1995 and 2014. By applying Blinder-Oaxaca decomposition to the concentration index decomposition equations, changes in the concentration index between the 2 years can be formulated as follows:

$$ {\mathrm{C}\mathrm{I}}_{2014}-\mathrm{C}{1}_{1995}=\sum \limits_{\mathrm{k}}{\upeta}_{\mathrm{k},2014}\left({\mathrm{C}}_{\mathrm{k},2014}-{\mathrm{C}}_{\mathrm{k},1995}\right)+\sum \limits_{\mathrm{k}}{\mathrm{C}}_{\mathrm{k},1995}\left({\upeta}_{\mathrm{k},2014}-{\upeta}_{\mathrm{k},1995}\right), $$

$$ \Delta \ \mathrm{CI}=\sum \limits_{\mathrm{k}}{\upeta}_{\mathrm{k},2014}\ {\Delta  \mathrm{C}}_{\mathrm{k}}+\sum \limits_{\mathrm{k}}{\mathrm{C}}_{\mathrm{k},1995}\ {\Delta  \upeta}_{\mathrm{k}}, $$

where *∆* CI indicates the change in the concentration index of antenatal care between 1995 and 2014,

CI_1995_and C1_2014_: the concentration index for the antenatal care for 1995 and 2014, respectively,

η_k, 1995_ and η_k, 2014_: the elasticity of the determinant (k) for 1995 and 2014, respectively, ∆η_k_ indicates the change in the elasticity of the determinant (k) between 1995 and 2014,

C_k, 1995_ *and* C_k, 2014_: the concentration index of the health determinant for 1995 and 2014, respectively, and.

∆C_k_ indicates the change in the concentration index of the determinant (k) between 2014 and 1995.

Accordingly, the contribution of any determinant xk to the change in antenatal care use inequality (*∆* CI) equals:

$$ \Delta \ {\mathrm{C}\mathrm{I}}_{\mathrm{k}}={\upeta}_{\mathrm{k},2014}\left({\mathrm{C}}_{\mathrm{k},2014}-{\mathrm{C}}_{\mathrm{k},1995}\right)+{\mathrm{C}}_{\mathrm{k},1995}\left({\upeta}_{\mathrm{k},2014}-{\upeta}_{\mathrm{k},1995}\right) $$

Finally, to provide some assessment of the relative importance of the inequality versus the elasticity component of *∆* C_k_, two components were calculated for each health determinant, namely the relative share of the change of its elasticity C_k, 1995_(η_k, 2014_ − η_k, 1995_)/*∆* CI_k_, and the relative share of the change of its inequality η_k, 2014_(C_k, 2014_ − C_k, 1995_)/*∆* CI_k_.

## Results

### Antenatal care and its determinants

Table 1 presents a profile and a comparison of antenatal care visits in Egypt in the two surveys and their main determinants. It shows a significant improvement in the use of antenatal care services: while only 41.7% of women had had at least one antenatal care visit in 1995, this proportion had increased to 90.2% by 2014. A similar improvement is observed for URAC: while just 30.4% of women had received URAC in 1995, that number had increased to 89.2% of women in 2014.

**Table 1:** Percentage distribution and concentration indexes for URAC and its determinants (1995 and 2014)

Variables	1995		2014	
	%	CI	%	CI
**Antenatal care**				
Any antenatal care***	41.7	0.490	90.20	0.101
Regular antenatal care***	30.4	0.507	82.90	0.172
Age at birth ***				
< 20 years	10.00	−0.146	7.40	−0.119
20-	27.40	−0.018	30.50	0.004
25-	29.10	0.067	32.40	0.030
30-	18.90	0.026	19.10	0.010
35-	10.70	−0.016	8.60	− 0.027
40+	3.90	−0.079	2.00	−0.080
Respondent’s educational attainment***				
No education	44.30	−0.352	18.30	−0.393
Primary	22.00	−0.019	8.90	−0.205
Secondary	28.10	0.406	57.30	0.036
Higher	5.70	0.812	15.60	0.446
Respondent’s husband’s educational attainment***				
No education	27.80	−0.407	13.20	−0.286
Primary	28.00	−0.130	13.60	−0.133
Secondary	33.40	0.235	56.60	−0.009
Higher	10.90	0.654	16.60	0.365
Region ***				
Urban governorate	18.00	0.475	10.50	0.712
Urban Lower Egypt	10.30	0.430	9.30	0.616
Rural Lower Egypt	30.00	−0.124	39.40	− 0.154
Urban Upper Egypt	11.30	0.307	11.10	0.479
Rural Upper Egypt	29.40	−0.437	28.80	−0.438
Frontier governorate	1.00	0.142	1.00	0.123
Toilet type (modern)***	21.60	0.732	52.30	0.350
Experience of terminated pregnancy ***	26.70	−0.022	20.70	−0.066
**Birth attributes**				
Birth interval > 24 months)***	78.80	0.027	84.50	0.011
Single birth**	98.40	0.001	97.80	0.001
Birth order ***	3.90	−0.115	2.70	− 0.074
**Previous birth experience**				
Delivery at medical unit***	11.00	0.250	28.60	0.012
Delivered by C-section***	1.90	0.328	15.80	0.085
Live birth	75.00	−0.008	75.80	−0.028

*** Significant < 0.001, ** Significant < 0.01

This improvement in use of antenatal care was accompanied by significant changes in the demographic attributes of the women surveyed. Regarding age at birth, the proportions of women who gave birth at both young and old ages decreased significantly between the two surveys; while 75.4% of women gave birth between the age of 20 and 35 in 1995, this proportion increased to 82% in 2014. The majority of the declines occurred among older women (35 and over).

On the social front, in 2014 the level of educational attainment achieved by women and their husbands showed significant gains compared to the levels in 1995. In 2014, almost 73% of women had secondary or higher education, compared to 34% in 1995. Similarly, while only 44.3% of the women’s husbands had secondary or higher education in 1995, this proportion had increased to 73.2% in 2014.

Using the lack of a modern toilet facility as a proxy for household standard of living, Table 1 shows significant improvement in these standards of living, with the proportion of households with a modern toilet increasing from 21.6% in 1995 to 52.3% in 2014.

On the health needs front, except for the death of the previous child and having had multiple births, Table 1 shows significant improvement on all indicators. Experience of terminated pregnancies among the sample decreased significantly, from 26.7% in 1995 to 20.7% in 2014. The prevalence of appropriate preceding birth interval (more than 24 months) increased from 78.8% in 1995 to 84.5% in 2014. Birth order also showed a significant decrease between 1995 and 2014, from 3.9 to 2.7. Delivery in medical centers also increased from 11% in 1995 to 28.6% in 2014. By contrast, the prevalence of cesarean section increased from 1.9% in 1995 to 15.8% in 2014.

Improvement in the prevalence of URAC was accompanied by significant improvement/reduction in the levels of wealth-based inequality; while in 1995 CI for URAC was 0.507, it had declined to 0.172 in 2014, a reduction of 66.1%.

Table 1 also shows the concentration indexes for the main determinants of URAC. In 1995, wealth-related inequalities were large for almost all the determinants. On one hand, women aged below 25 years and those 35 years or older while giving birth, those with low levels of education, low levels of husband’s education, and rural residents of both Lower Egypt and Upper Egypt, were highly concentrated among relatively poor respondents. The factor of a large number of children was also highly concentrated among poor respondents. On the other hand, proper birth intervals, delivery in medical units and cesarean section were less prevalent among relatively poor respondents.

In 2014, similar patterns of the wealth-related inequalities in the determinants of URAC can be observed, with the majority of the inequalities declining in magnitude, reflecting improvements in their wealth-related differentials. For example, while CI for the respondents’ secondary education was 0.406 in 1995, in 2014, CI had decreased to 0.036. Similarly, wealth-related differentials in birth order declined from CI = -0.115 in 1995 to CI = -0.066 in 2014. The concentration of the poor who live in rural Upper Egypt showed a stability between the 2 years (CI = -0.438). By contrast, an increase in inequality was observed in the experience of terminated pregnancies, and for respondents living in urban Lower and Upper Egypt.

### Determinants of use of regular antenatal care

Table 2 presents the odds ratios for the logit coefficients for URAC in the years 1995 and 2014. For 1995, older age at birth and high levels of education in both the respondent and her husband were significantly more likely to correlate with URAC. Regional patterns for URAC exhibited lower odds of use among all regions, in particular the rural regions. For example, living in rural Upper Egypt decreased the odds of URAC by 71%.

**Table 2:** Odds ratio for use of antenatal care in 1995 and 2014

Variables	1995	2014	
**Age at birth**			
20-	1.21	1.05	
25-	1.73***	1.24	
30-	1.93***	1.43	*
35-	1.98***	1.34	
40+	2.19**	1.84	*
**Respondent’s educational attainment**			
Primary	1.38**	1.11	
Secondary	2.54***	1.51	***
Higher	4.68***	2.61	***
**Respondent’s husband’s educational attainment**			
Primary	1.40**	1.39	**
Secondary	1.44**	1.51	***
Higher	1.92***	1.95	***
**Region**			
Urban Lower Egypt	0.89	0.99	
Rural Lower Egypt	0.43***	0.82	
Urban Upper Egypt	0.74*	0.59	***
Rural Upper Egypt	0.29***	0.48	***
Frontier governorate	0.43***	0.52	***
**Toilet type (modern)**	2.47***	1.20	*
**Experience of terminated pregnancy**	1.73***	1.26	**
**Birth attributes**			
Birth interval > 24 months	1.46***	1.46	***
Single birth	0.78	0.58	*
Birth order	0.89***	0.83	***
**Previous birth experience**			
Delivery at medical unit	2.16***	0.93	
Delivered by C-section	0.98	1.84	***
Live birth	0.55***	0.45	***
Pseudo R^2^	0.26	0.11	
Log likelihood	− 3317.39	− 4451.88	

*** Significant < 0.001, ** Significant < 0.01, *Significant at < 0.05

Proper preceding birth intervals were associated with significantly higher odds of URAC, while a higher birth order was significantly related to lower odds of regular use of antenatal care (OR = 0.89). Delivery in a medical unit for the previous birth was also associated with 2.16 higher odds of regular use of antenatal care. A previous live birth decreased the odds of URAC by 45%.

In 2014, the odds of URAC showed a similar pattern as in 1995, but the effects of the different determinants on URAC were attenuated. For age at birth, only those giving birth aged 30–35, or over 40, exhibited higher odds of use compared to other age groups. Education for both the respondents and their husbands continued to be positively related to increased odds of URAC. Residents of regions other than urban governorates and urban Lower Egypt showed lower odds of URAC. High standards of living were also associated with 26% higher odds of antenatal care. Delivery of a previous birth by cesarean section showed 84% increases in URAC.

### Decomposition of use of regular antenatal care inequality

**Fig. 1:**
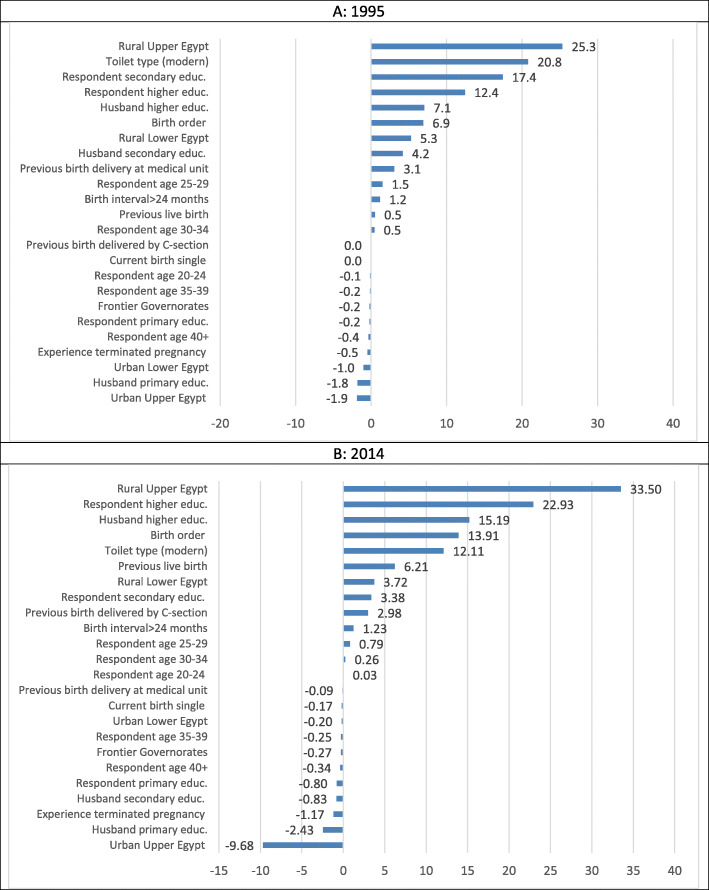
Decomposition share of the inequalities in the determinants of URAC (1995 and 2014)

Table 3 presents the results of the decomposition of the inequalities in URAC into its determinants. Figure 1a and b show the different shares of the inequality in the determinants of receiving regular antenatal care in 1995 and 2014, respectively.

**Table 3:** Decomposition of the wealth-related inequalities in URAC

Variables	2014						1995					
	dy/dx	CI	Mean	Elasticity	Share	% Share	dy/dx	CI	Mean	Elasticity	Share	% Share
**Age at birth**												
20-	0.007	0.004	0.305	0.003	0.0000	0.0	0.025	− 0.018	0.274	0.023	−0.0004	− 0.1
25-	0.029	0.030	0.324	0.011	0.0003	0.8	0.078	0.067	0.291	0.074	0.0050	1.5
30-	0.046	0.010	0.192	0.011	0.0001	0.3	0.094	0.026	0.189	0.058	0.0015	0.5
35-	0.038	−0.027	0.086	0.004	−0.0001	− 0.3	0.098	− 0.016	0.107	0.034	−0.0005	− 0.2
40+	0.073	−0.080	0.020	0.002	−0.0001	−0.3	0.114	−0.079	0.039	0.015	−0.0012	− 0.4
**Respondent’s educational attainment**												
Primary	0.015	−0.205	0.089	0.002	−0.0003	−0.8	0.048	−0.019	0.220	0.035	−0.0007	−0.2
Secondary	0.057	0.036	0.573	0.040	0.0014	3.4	0.153	0.406	0.281	0.142	0.0575	17.4
Higher	0.115	0.446	0.156	0.022	0.0096	22.9	0.271	0.812	0.057	0.051	0.0410	12.4
**Respondent’s husband’s educational attainment**												
Primary	0.047	−0.133	0.136	0.008	− 0.0010	−2.4	0.050	−0.130	0.279	0.046	−0.0059	−1.8
Secondary	0.057	−0.009	0.566	0.039	−0.0003	− 0.8	0.054	0.235	0.334	0.059	0.0139	4.2
Higher	0.087	0.365	0.166	0.017	0.0064	15.2	0.099	0.654	0.109	0.036	0.0233	7.1
**Region**												
Urban Lower Egypt	−0.001	0.616	0.093	0.000	−0.0001	− 0.2	−0.023	0.430	0.103	−0.008	− 0.0033	−1.0
Rural Lower Egypt	− 0.021	− 0.154	0.394	− 0.010	0.0016	3.7	−0.142	− 0.124	0.300	− 0.140	0.0174	5.3
Urban Upper Egypt	−0.064	0.479	0.111	−0.008	−0.0041	−9.7	− 0.054	0.307	0.113	−0.020	− 0.0062	−1.9
Rural Upper Egypt	−0.093	− 0.438	0.287	− 0.032	0.0141	33.5	− 0.198	− 0.437	0.294	− 0.191	0.0836	25.3
Frontier governorate	−0.081	0.123	0.009	−0.001	− 0.0001	− 0.3	− 0.142	0.142	0.010	− 0.005	− 0.0007	− 0.2
**Toilet type (modern)**	0.023	0.350	0.523	0.015	0.0051	12.1	0.132	0.732	0.216	0.094	0.0686	20.8
**Experience of terminated**												
**pregnancy**	0.030	−0.066	0.207	0.007	− 0.0005	−1.2	0.080	− 0.022	0.267	0.070	− 0.0016	−0.5
**Birth attributes**												
Birth interval > 24 months)	0.048	0.011	0.845	0.049	0.0005	1.2	0.055	0.027	0.788	0.142	0.0039	1.2
Single birth	−0.069	0.001	0.978	−0.081	− 0.0001	− 0.2	− 0.036	0.001	0.984	− 0.117	− 0.0001	0.0
Birth order	−0.024	− 0.074	2.675	− 0.079	0.0058	13.9	−0.016	− 0.115	3.714	− 0.199	0.0228	6.9
**Previous birth experience**												
Delivery at medical unit	− 0.009	0.012	0.286	−0.003	0.0000	−0.1	0.113	0.250	0.110	0.041	0.0102	3.1
Delivered by C-section	0.077	0.085	0.158	0.015	0.0013	3.0	−0.004	0.328	0.019	0.000	−0.0001	0.0
Live birth	−0.103	− 0.028	0.758	− 0.094	0.0026	6.2	−0.087	−0.008	0.750	−0.215	0.0017	0.5

Figure 1a reveals that, in 1995, six determinants of URAC accounted for 90% of its inequality. In 1995, these determinants (arranged by their relative share) are: living in rural Upper Egypt (25.3%), household standard of living indicator (20.8%), women’s secondary or higher education (17.4 and 12.4%, respectively), husbands with higher education (7.1%), and birth order of the child (6.9%). In other words, 90% of the wealth based inequality in URAC would decrease either if living in rural Upper Egypt, household standard of living, women’s secondary or higher education, husband’s with higher education and child birth order become equally distributed across the wealth groups or if their regression coefficients would become zero.

Figure 1b shows that, in 2014, five the previous six determinants continued to play a major role in explaining the inequalities in URAC and account for 97.6% of the inequalities. The highest share was attributed to inequalities in living in rural Upper Egypt (33.5%), followed by inequalities in the respondent’s higher education (22.9%) and husband’s higher education (15.2%). Inequalities in birth order accounted for 13.9% and household standard of living retreated, accounting for 12.1% of the inequality in antenatal care. In other words, 97.6% of the wealth based inequality in URAC would decrease either if living in rural Upper Egypt, household standard of living, women’s secondary or higher education, husband’s with higher education and child birth order become equally distributed across the wealth groups or if their regression coefficients would become zero.

Changes in the determinants’ shares between 1995 and 2014 reveal the role played by their concentration indexes and elasticities. For example, the analysis shows that living in rural Upper Egypt maintained its place as providing the largest contribution to inequality in URAC in both years. In 1995, this large share was mainly the product of a high concentration of poor women in this region (CI = -0.437) and its large elasticity in URAC (η = − 0.191), resulting in a 25.3% share. Between 1995 and 2014, this share increased to 33.5%. This increase was the product of the lower value CI for URAC in 2014 compared to the level in 1995. At the same time, living in rural Upper Egypt maintained its high concentration of poor women (CI = -0.438), but experienced some improvement in its elasticity (η = − 0.093). For household standard of living (toilet type), the 20.8% share in 1995 was the product of its high concentration among the rich (CI = 0.732) and relatively moderate elasticity (η =0.093). By contrast, in 2014, the concentration index declined by almost 50% (CI = 0.350) and elasticity also decreased, reaching (η =0.015), producing a decline in its share to 12.1%. The respondent’s higher education showed an increase in its share between 1995 and 2014 from 12.4 to 22.9%, respectively, despite the improvement in its concentration index (from 0.812 to 0.446) and elasticity (from 0.051 to 0.022).

### Decomposition of the change in the wealth-related inequalities of use of regular antenatal care between 1995 and 2014

Understanding the main source for the decline in wealth-related inequalities in URAC is an important exercise to assess the contribution of the different policies over the period under consideration. Table 4 shows the changes in the determinants of URAC that contributed to the decline in its inequality between 1995 and 2014. These changes are further divided between its two components, namely changes in their elasticity (η_k_) and changes in their concentration index (C_k,_). The assessment of a determinant’s contribution to the change in the inequalities in regular use of antenatal care is carried out in terms of the overall sign and the magnitude of this contribution. A large absolute value indicates a large contribution. As the difference in the inequality of URAC is assessed in terms of inequality in 2014 minus inequality in 1995, a positive sign for any determinant indicates that this determinant has contributed to the decline in inequality, while a negative sign indicates that this determinant has counteracted the decline in the inequality.

**Table 4:** Decomposition of changes in the wealth-related inequalities in URAC

Variables	Differences in		Share			% Share		
	Elasticity	CI	Elasticity	CI	Total	Elasticity	CI	Total
**Age at birth**								
20-	−0.020	0.022	0.000	0.000	0.000	−0.1	0.0	−0.15
25-	−0.063	− 0.038	− 0.002	−0.003	− 0.005	0.6	1.0	1.62
30-	−0.048	− 0.016	0.000	−0.001	− 0.001	0.2	0.3	0.48
35-	−0.030	−0.011	0.001	0.000	0.000	−0.3	0.1	−0.15
40+	−0.013	− 0.001	0.001	0.000	0.001	−0.4	0.0	−0.36
**Respondent’s educational attainment**								
Primary	−0.033	−0.185	0.007	−0.006	0.000	−2.4	2.3	−0.12
Secondary	−0.102	−0.370	− 0.004	−0.052	− 0.056	1.3	18.2	19.49
Higher	−0.029	−0.366	− 0.013	−0.019	− 0.031	4.5	6.4	10.91
**Respondent’s husband’s educational attainment**								
Primary	−0.038	−0.003	0.005	0.000	0.005	−1.7	0.0	−1.71
Secondary	−0.020	−0.243	0.000	−0.014	− 0.014	−0.1	5.0	4.94
Higher	−0.018	−0.288	− 0.007	−0.010	− 0.017	2.3	3.6	5.86
**Region**								
Urban Lower Egypt	0.007	0.186	0.005	−0.001	0.003	−1.6	0.5	− 1.11
Rural Lower Egypt	0.130	−0.030	−0.020	0.004	−0.016	7.0	−1.5	5.50
Urban Upper Egypt	0.012	0.172	0.006	−0.003	0.002	−1.9	1.2	−0.74
Rural Upper Egypt	0.159	−0.001	−0.070	0.000	−0.070	24.2	−0.1	24.16
Frontier governorate	0.004	−0.019	0.000	0.000	0.001	−0.2	0.0	−0.19
**Toilet type (modern)**	−0.079	−0.382	− 0.028	−0.036	− 0.064	9.6	12.4	22.08
**Experience of terminated pregnancy**	−0.063	−0.044	0.004	−0.003	0.001	−1.4	1.1	−0.37
**Birth attributes**								
Birth interval > 24 months)	−0.093	−0.017	− 0.001	−0.002	− 0.003	0.3	0.8	1.17
Single birth	0.036	0.000	0.000	0.000	0.000	0.0	0.0	−0.01
Birth order	0.120	0.040	−0.009	−0.008	− 0.017	3.1	2.8	5.89
**Previous birth experience**								
Delivery at medical unit	−0.044	−0.237	− 0.001	−0.010	− 0.010	0.2	3.4	3.55
Delivered by C-section	0.015	−0.243	0.001	0.000	0.001	−0.4	0.0	−0.46
Live birth	0.122	−0.020	−0.003	0.004	0.001	1.2	−1.5	−0.30

**Fig. 2:**
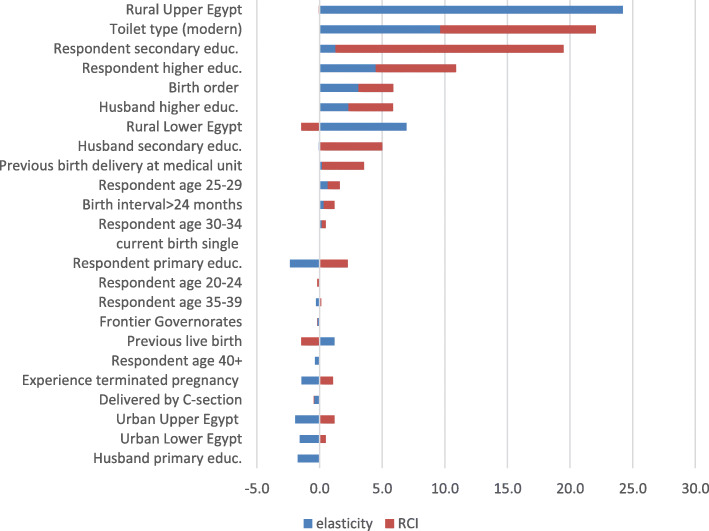
Share of the changes in the determinants of inequalities and elasticities in the changes in the inequalities in URAC (1995 and 2014)

Figure 2 shows that socioeconomic determinants were the largest contributors to the decline in wealth-based inequality in URAC between 1995 and 2014. Of the 12 determinants with positive signs, nine were socioeconomic determinants. Living in rural Upper Egypt, the household’s standard of living, and the woman’s attainment of secondary or higher education were the largest contributors to the decline in inequality, with a total share of 76.6% (24.1, 22.1, 19.5 and 10.9%, respectively).

These were followed by husbands’ higher or secondary education and living in rural Lower Egypt, which accounted for a total share of 16.3% (5.9, 4.9 and 5.5%, respectively). By contrast, only three health need determinants showed positive contributions to the decline in URAC, namely birth order (5.9%), delivery in a medical center (3.6%) and appropriate birth interval (> 24 months) (1.2%).

Examination of the two components of the determinant’s contribution to the decline in inequality revealed different patterns for different determinants. Table 4 shows that the contributions of living in rural Upper Egypt and living in rural Lower Egypt were mainly due to declines in elasticity between 1995 and 2014. By contrast, the contribution of a woman’s and her husband’s secondary education was mainly due to declines in CI during the same period. For the other positively contributing determinants, their contributions were divided between declines in elasticity (η) and declines in CI, with the share of the declines in CI commonly larger than that for elasticity. For example, the 22.1% contribution of the household standard of living was divided into 12.4% for declines in CI and 9.7% for declines in elasticity. Similarly, the 10.9% contribution of women’s higher education was divided into 6.4% for declines in CI and 4.5% for declines in elasticity.

## Discussion

Over a period of 20 years, Egypt successfully increased URAC from 30.4 to 82.9%. At the same time, Egypt managed to decrease its wealth-based inequalities in URAC, with the difference between the richest and poorest wealth quintiles declining from 61% in 1995 to 21.1% in 2014. The current paper investigates the changes in the main determinants that contributed to the wealth-based inequality in URAC for the 2 years 1995 and 2014, as well as the main determinants that underlined the declines in this inequality in this period.

These changes in wealth disparities were confirmed by the decline in the wealth-based CI from 0.507 in 1995 to 0.172 in 2014. This decline was also associated with improvements in the inequalities of many of its determinants, in particular the ages of childbearing, women’s and their husbands’ education, household standard of living, and birth order. Nevertheless, other determinants either maintained the same level of inequalities, such as living in rural Upper Egypt, or showed increases in inequalities, such as experience of a terminated pregnancy and living in urban Lower or Upper Egypt.

As modeling is the common approach in assessing the relationship between health variables and their determinants, modeling URAC for the 2 years of 1995 and 2014 was carried out. The modeling of URAC confirmed the findings in previous literature on developing countries [12–29]: both social and health needs were significant in determining URAC. On the social side, older age at birth, less than secondary education for women and their husbands, low household standard of living/wealth, and living in rural Upper Egypt (the most conservative and underdeveloped region of the country) were significantly associated with lower odds of URAC than their comparative social groups. These findings are consistent with findings from many similar studies in developing countries including Pakistan [29], Nigeria [26], Ethiopia [21], Nepal [16], Sudan [12] and earlier studies in Egypt [19]. Previous research in Egypt showed that women with at least secondary education showed higher odds of using any antenatal care services than those with less than secondary education [19}. The current study also showed that older age at birth was associated with higher odds of URAC in comparison to women who give birth at young. The same finding was observed in Ethiopia [21]. Our finding that women living in rural Upper Egypt showed the lowest odd of URACg was also consistent with findings in previous studies in Egypt [19, 27.34}.

Changes in the relationship between URAC and its determinants confirmed the findings of previous literature with regard to the effect of health needs in particular birth order on use of antenatal care [12–34]. High birth order was associated with lower odds of use of full antenatal care in rural India [29], Pakistan [28], Ethiopia [21] and Nepal [16]. It was also significantly associated with delayed use of antenatal care in Rwanda [15]. In Sudan, parity equal or greater than 3 was associated with double the odds of antenatal care inadequacy [12]. One possible explanation for this lower odds of URAC is women’s high confidence and experience gained after delivering their first birth and their tendency to use their accumulated experience and knowledge from previous pregnancies [44]. This same reason also can explain the study finding that showed high odds of using URAC with the experience of terminated pregnancy and/or the death of the previous birth. In these two incidences, women loss confidence in their experience and become more cautious about their pregnancies and hence maintained regular visit to antenatal care services. Similar findings were reported by Zaki and colleagues in examining antenatal care for the 2 years 2000 and 2008 [19].

The main contribution of the current study is the introduction of the decomposition analysis of the inequalities in URAC and the changes in these inequalities between 1995 and 2014. While the modeling approach leads to the identification of the significant relationships between URAC and its determinants, recommendations based on this approach commonly focus on strengthening the health system to better serve the population, or blaming individuals for their underutilization of URAC. By contrast, decomposition of the inequalities quantifies two factors underlying URAC inequality, namely: 1) the relationship between URAC and its determinants assessed in terms of the determinants’ elasticity; and 2) the inequality in these determinants assessed in terms of determinants’ CI. While the elasticity can be interpreted as the success of the health system in expanding its services to all individuals, CI shows the role of inequality in the determinants. The decomposition of URAC inequality for the 2 years showed the substantial contribution of five main determinants that accounted for more than 90% of the estimated inequality for both years. These determinants were dominated by social determinants, except for birth order. The social determinants were: living in rural Upper Egypt, the respondent’s higher education, the husband’s higher education, and the household standard of living. Similar findings were observed in Rural India [29] and Nigeria [26] in which inequalities in the social determinants of antenatal care utilization accounted for more than 80% of its wealth based inequality. In rural India, the social determinants of use of full antenatal care accounted for more than 80% of its wealth based inequality [29]. However, the study also showed that the relative contributions of these social determinants changed in the time between the 2 years. These changes in relative contributions were the product of the interplay between three changes, namely the decline in the concentration indexes for URAC, the decline in the concentration indexes in the majority of the determinants, and the changes in the elasticities of these determinants.

This interplay was more redefined in the decomposition of the changes in the inequalities of URAC between 1995 and 2014. In this decomposition, the above five determinants were the most-contributing determinants to the decline in the URAC inequalities. As the determinant’s contribution is the product of the sum of the changes in its concentration index and the changes in its elasticity, declines in concentration index indicate the success of social policies in decreasing inequality of the social determinants and the success of the health system in decreasing inequality of health need determinants. By contrast, declines in elasticity indicate the success of health system in attenuating the effect of the determinant on URAC. With this understanding, areas for interventions can be identified. For example, living in rural Upper Egypt was the biggest contributor to the inequalities and the biggest contributor to the changes in the inequalities; analysis revealed that the decline in URAC inequality was mainly due to the changes in the elasticity of this determinant, which can be traced to the better performance of the health system, attenuating its effect on URAC. This confirmed the results of Gipson and colleagues that showed that the focus of government efforts on improving the maternal service in most vulnerable areas including rural Upper Egypt have paid off in the form of significant declines in their maternal mortality ratio [9]. By contrast, there was no change in its concentration index. In other words, despite the ongoing effort to tackle poverty in rural Upper Egypt, poverty continues to be significantly prevalent in this region. This result is in line with the findings from the Egypt Household Income, Expenditure, and Consumption Survey in 2017, which showed that the high concentration of poverty in this region did not change between 2005 and 2015, with more than 50% of the population classified as poor [45]. The result in the current analysis highlights the need for an area of intervention at the level of social policies to tackle these high levels of poverty, which in turn could lead to more declines in URAC inequalities.

The large shares of the women’s and husband’s education in the changes in URAC inequalities can be attributed to declines in their concentration index, which confirm the observed improvement in educational attainment of women and men in Egypt in that time period. UN data show that the proportion of Egyptians with at least secondary education among those aged 25 years and older increased from 29.7% in 1995 to 61.4% in 2014 [46]. However, the changes in their elasticity were relatively small, which could point to the need for the health system to direct more interventions to attenuating the impact of women’s and their husbands’ education on URAC. For household standards of living, there is still more area for intervention, particular on the level of the social policies to decrease its inequalities. Similarly, for other positively contributing determinants, the declines in the concentration index and, to a lesser extent, in the elasticity, showed the need to work more on interventions in the health system and in social policies.

As any scientific research study, the current study has certain limitations. First, although the study used data for the latest available Egyptian DHS, the 2014 survey is unable to account for the current situation of the antenatal care services. Second, the study main focus was on the individual behavior of women. The study did not assess any of the antenatal care services-related factors including their availability, accessibility, affordability and quality as well as overall community attributes that are related to antenatal care. Finally, the cross-section nature of the data used in the current study does not permit the establishment of causality.

## Conclusion

Wealth based inequality in the URAC has declined significantly between 1995 and 2014. This decline was also associated with relative improvement in its social determinants inequalities. Nevertheless, poverty still wide spread in rural Upper Egypt, wealth inequality in secondary education still exist and poor people maintain their relatively high parity. These social factors continue to play a major role in determining URAC, its inequality and changes overtime. Future policies and intervention should direct specific attention to these social factors and adopt social initiatives with an equity lens that will not *leave no one behind.* These policies should focus on exploring the structural causes for the inequalities in these factors and develop needed social initiatives to address them. These efforts should also focus on the tackling inequalities in the health system among the different social groups.

In addition, the current study offers clear insight into the use of decomposition in tackling health inequality in general. Decomposition of inequality can support prioritization of interventions and hence can lead to effective and efficient use of the limited resources available in any society. Decomposition of the changes in inequality can aid assessment of the effectiveness and the monitoring of the impact of these interventions, as well as help generate new policies and interventions needed to tackle these inequalities.

## Data Availability

The dataset analyzed during the current study is available in The DHS program: The Demographic and Health Survey program repository. https://www.dhsprogram.com/data/dataset/Egypt_Standard-DHS_2014.cfm?flag=0

## Abbreviations

URAC: Utilization of regular antenatal care
CI: Concentration index
DHS: Demographic and health survey
EDHS: Egypt Demographic and Health Survey
CSDH: Commission on Social Determinants of Health
